# Serum albumin at resection predicts in-hospital death, while serum lactate and aPTT on the first postoperative day anticipate anastomotic leakage after Ivor-Lewis-esophagectomy

**DOI:** 10.1007/s00423-022-02510-y

**Published:** 2022-04-28

**Authors:** Florian Scheufele, Thomas Vogel, Melanie Gasiorek, Alexander Novotny, Helmut Friess, Ihsan Ekin Demir, Stephan Schorn

**Affiliations:** Department of Surgery, Klinikum Rechts Der Isar, School of Medicine, Technical University of Munich, Ismaningerstrasse 22, 81675 Munich, Germany

**Keywords:** Ivor-Lewis-esophagectomy, Death, Serum lactate, aPTT, Anastomotic leakage, Morbidity, Mortality

## Abstract

**Background:**

Anastomotic leakage (AL) is a major complication after esophagectomy, potentiating morbidity and mortality. There are several patient risk factors associated with AL, but high-fidelity postoperative predictors are still under debate. The aim was to identify novel reliable predictors for AL after esophagectomy.

**Methods:**

A high-volume single-center database study, including 138 patients receiving Ivor-Lewis-esophagectomy between 2017 and 2019, was performed. Serum levels of albumin, aPTT, and lactate before and after surgery were extracted to assess their impact on AL and in-hospital mortality.

**Results:**

High serum lactate on postoperative day 1 (POD1) could be shown to predict AL after esophagectomy [AL vs. no AL: 1.2 (0.38) vs. 1.0 (0.37); *p* < 0.001]. Accordingly, also differences of serum lactate level between end (POD0-2) and start of surgery (POD0-1) (p < 0.001) as well as between POD1 and POD0-1 (*p* < 0.001) were associated with AL. Accordingly, logistic regression identified serum lactate on POD 1 as an independent predictor of AL [HR: 4.37 (95% CI: 1.28–14.86); *p* = 0.018]. Further, low serum albumin on POD0 [2.6 (0.53) vs. 3.1 (0.56); *p* = 0.001] and high serum lactate on POD 0–1 [1.1 (0.29) vs. 0.9 (0.30); *p* = 0.043] were associated with in-hospital death. Strikingly, logistic-regression (HR: 0.111; *p* = 0.008) and cox-regression analysis (HR: 0.118; *p* = 0.003) showed low serum albumin as an independently predictor for in-hospital death after esophagectomy.

**Conclusions:**

This study identified high serum lactate as an independent predictor of AL and low serum albumin as a high-fidelity predictor of in-hospital death after esophagectomy. These parameters can facilitate improved postoperative treatment leading to better short-term as well as long-term outcomes.

## Introduction

Malignancies of the esophagus with an incidence of 13.3 per 100 000 for men and 3.8 per 100 000 for women represent a significant tumor burden to patients [1]. For a curative treatment, radical esophagectomy with or without neoadjuvant therapy is a crucial aspect of treatment. Ivor-Lewis esophagectomy, with its thoracic and abdominal approach, represents one of the most challenging resections in visceral surgery [2]. After esophagectomy, anastomotic leakage (AL) with an incidence of 5–30% plays a critical role, as it significantly influences postoperative morbidity (77.5% vs. 47.3%) length of hospital stay (23 vs. 11 days) as well as in-hospital mortality (12.3% vs. 3.8%) [3–5]. This is caused by the development of sepsis due to mediastinitis and peritonitis as well as respiratory failure with the need for reintubation, pneumonia, or atrial fibrillation and the need for reoperation [6, 7]. Furthermore, AL also negatively influences long-term oncological outcomes after esophagectomy with decreased overall survival and quality of life as well as earlier tumor recurrence [8]. Also, up to 40% of patients suffering from AL after esophagectomy require endoscopic dilatations due to the formation of stenosis [9, 10].

Therefore, it is warranted to reduce the incidence of AL after esophagectomy to improve the patient’s overall outcome. In this context, cardiovascular risk factors like arterial hypertension or former ischemic heart disease have already been linked to the development of AL [8]. Also, the presence of diabetes mellitus, weight loss, preoperative serum albumin levels, forced expiratory volume (FEV1) < 2 L, respiratory complications, and intraoperative blood loss have been shown to be significant risk factors for AL [7, 11]. As some of these risk factors cannot be avoided, early detection of AL and identification of patients at risk is a crucial point for postoperative care. This facilitates timely initiation of treatment of AL and thus can confer to amelioration of complications within the further postoperative course. AL can be treated conservatively, endoscopically, or surgically, although the endoscopic treatment is favored in light of reduced morbidity and mortality [12]. Endoscopic treatment options include the insertion of a self-expanding metal stent or endoscopic vacuum therapy, both representing valuable options [13, 14]. To improve outcomes for patients with AL after esophagectomy, early start of treatment especially with endoscopic vacuum therapy seems to be crucial in ameliorating postoperative complications [15]. Thus, we sought to discover novel parameters to identify patients at risk for the development of AL after esophagectomy for facilitating timely postoperative treatment of AL. In this context, the leading mechanism of failure of the anastomotic healing is ischemia of the gastric tube due to decreased perfusion as well as technical failure [16]. Therefore, it sounds plausible that a marker of ischemic events like serum lactate might be a valuable marker to predict AL even in early stages.

## Patients and methods

### Data collection

The institutional surgical database was screened for all patients undergoing Ivor-Lewis esophagectomy between May 2017 and October 2019. Demographical characteristics including age and gender and comorbidities were gathered by screening electronic files and comorbidities were summarized using the Charlson comorbidity index [17]. Additional collected data included length of hospital stay (LOS), readmission within 30 days after discharge, postoperative complications including need for ICU, AL, date of detection of AL and postoperative complications according to Clavien-Dindo ≥ IIIa, cardiac complications, pulmonary complications, pulmonary embolism, neoadjuvant treatment, tumor stage, and in-hospital mortality. No restrictions were made regarding age and perioperative, oncological treatment. Only endoscopically confirmed AL was found to be clinically relevant and time of endoscopy was considered as the first diagnosis of AL. Routine endoscopy was performed on the 5^th^ postoperative day. The study was approved by the institutional review committee.

### Surgery

In this study, the operation technique was limited to Ivor-Lewis abdomino-thoracic en-bloc-esophagectomy with a right transthoracic approach and a gastric pull-up as previously described [2]: In brief, a transverse or median incision in the upper epigastrium followed by the partial division of the hiatus and mobilization of the lower portion of the esophagus was done. A gastric tube with a diameter of approx. 3 cm along the greater curvature was made after mobilization of the stomach using a linear stapler. All patients with malignant diseases received a D2-lymphadenectomy along the celiac axis and among the suprapancreatic region. Postoperative delayed gastric emptying was prevented in all patients by a transgastric dilatation of the pylorus for 5 s using a clamp. After closure of the abdomen, the position of the patient was changed from the prone position to the left lateral position. Following, a right-sided posterolateral thoracotomy and the en-bloc esophagectomy including the resection of the azygos vein, the ipsilateral pleura, and peri-esophageal tissue was performed. The transection line of the esophagus was above the azygos vein in all patients. Patients with malignant disease received a lower and middle mediastinal, subcarinal, and right-sided paratracheal lymphadenectomy. An end-to-side esophago-gastrostomy in the right pleural cavity was performed to reconstruct the gastrointestinal passage using a circular stapler. When a circular stapler was used for anastomosis, devices were inserted through the blind end of the gastric tube, which was closed after completion of the anastomosis using a linear cutter. Two thoracic drains, one drain located in the posterior mediastinum near the anastomosis and the other drain in the recessus of the diaphragm, were placed and the thoracotomy was closed [2, 18].

### Postoperative care

Epidural anesthesia was offered to all patients without contraindication. After the operation, extubation was performed in the operating room, and patients were transferred to an intermediate care until postoperative day (POD) 1. At POD 1, the clinical status of each patient was checked, and patients were transferred either to an intensive care unit or to a normal ward. A gastric tube was placed in all patients during the operation, which was removed until POD 3 followed by a start of oral liquid intake. Moreover, all patients received intensive mobilization and physiotherapy beginning from POD 1.

### Definition of anastomotic leaks

An esophagogastroduodenoscopy (EGD) was done in each patient at POD 5 ± 3 days regardless of the presence of symptoms or other serological markers [19]. A macroscopically visible necrosis of defect of the anastomosis was counted as an AL in accordance with the Esophagectomy Complications Consensus Group (ECCG) definition of AL [20]. Ischemias of the gastric tube were not included. In cases of detection of AL, further diagnostic procedures including CT were performed, and interventional drains were placed if necessary. If patients exhibited symptoms of AL, additional EGDs were performed to assess the degree of AL. Further, patients with AL, incidental or symptomatic, received placement of an endoluminal vacuum system, which was changed approximately every 3 days.

### Statistical analysis

All continuous variables were expressed as mean with standard deviation (SD) and compared using the *t* test. Categorical variables were expressed as frequency counts with corresponding percentages and differences between groups were assessed by the Fisher’s exact and chi-square test. Multivariate analysis for AL was done using the logistic regression analysis. The results of the regression analysis were expressed as hazard ratio (HR) with its corresponding 95%-CI and *p* value. Survival analyses were performed using Kaplan–Meier-survival curves, and differences were calculated using the long-rang test confirmed by cox-regression analysis. All statistical analyses were done using statistical software IBM SPSS, v25 for Windows (IBM Inc., USA).

For all comparisons, a two-sided *p* value was calculated and considered to be statistically significant for *p* value below 0.05.

## Results

### Demographic data

**Fig. 1:**
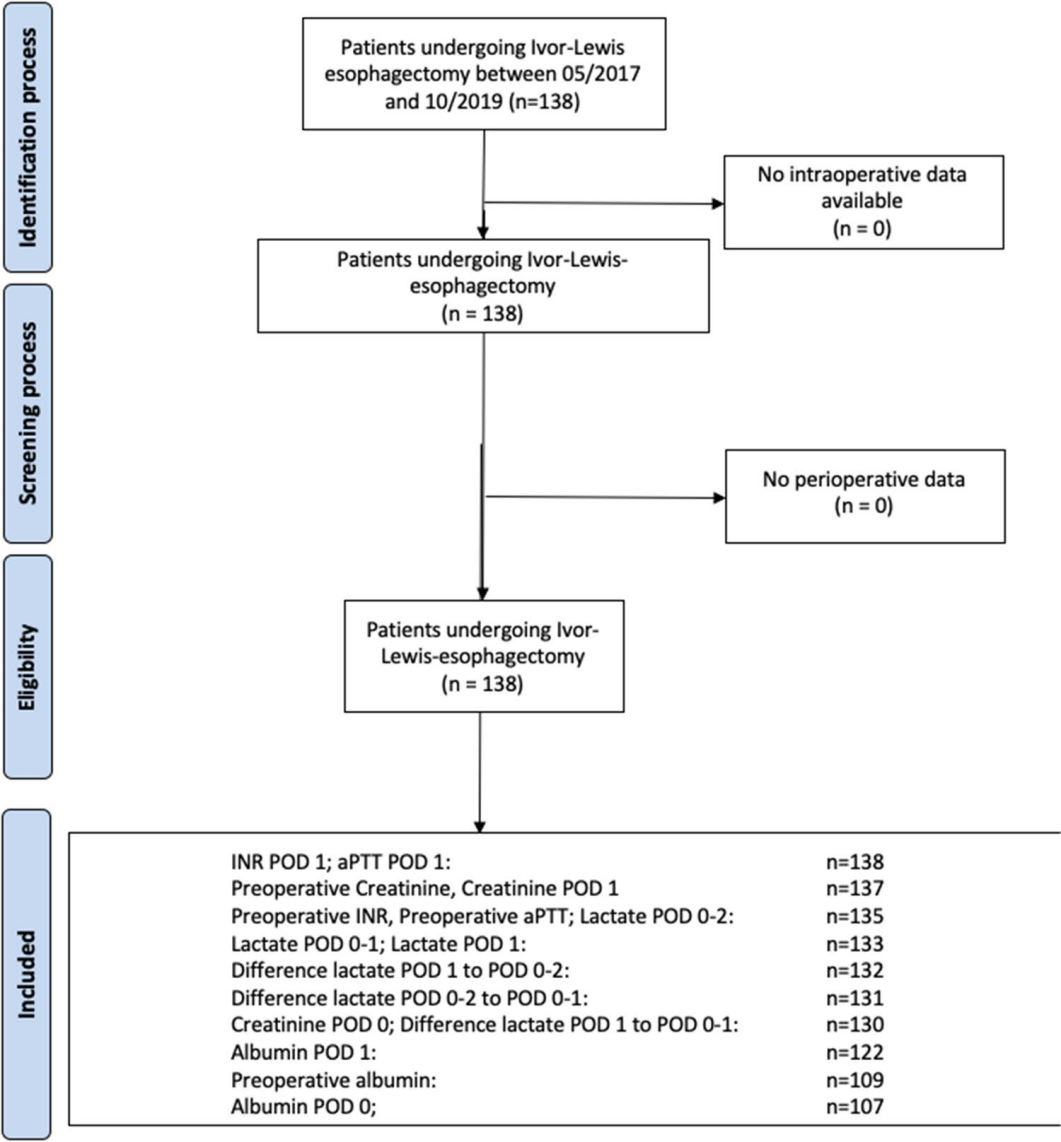
Flowchart of patient inclusion. POD (postoperative day), INR (international normalized ratio), aPTT (activated partial thromboplastin time)

Between 2017 and 2019, 138 patients receiving Ivor-Lewis esophagectomy were identified. Perioperative parameters were available in 138 patients for international normalized ratio (INR) and activated partial thromboplastin time (aPTT) on POD 1 and in 107 patients for albumin on POD 0 (Fig. 1). The mean age of included patients was 62.8 ± 11.1 years, and 80.4% were male (Table 1). The mean BMI was 25.4 kg/m2 (Table 1). Incidence of hypertension was 55.8%, of type II diabetes mellitus 13.0%, PAD 6.5%, coronary artery disease 18.8%, stroke 4.3%, and heart insufficiency 18.8% (Table 1). Of the included patients, 21.0% were classified ASA I, 34.1% ASA II, and 44.9% ASA III (Table 1). AEG I was present in 38.4%, AEG II in 28.3%, and AEG III in 1.4% of the patients, while 25.4% suffered from SCC of the esophagus (Table 1). Some 21.0% had T1 staged tumors, 21.7% T2 stage, 43.5% T3 stage, and 2.9% T4 stage. N0 was present in 57.2% of the patients, N1 in 17.4%, N2 in 10.1%, and N3 in 11.6% (Table 1). 91.3% had M0, while 4.3% of the patients had M1 stage (Table 1). Of the 138 patients analyzed, 105 (76.1%) did not have AL, while 33 (23.9%) developed AL after esophagectomy. Of those, 0 (0%) were type I, 30 (90.9%) were type II, and 3 (9.1%) were type III AL according to Esophagectomy Complications Consensus Group (ECCG) definition of anastomotic leakage [20]. Of 138 patients, 10 died during hospital stay corresponding to an in-hospital mortality rate of 7.2%. None of the demographical parameters were significantly different when comparing patients without AL and patients with AL (Table 1).

**Table 1:** Demographical data. Continuous variables are presented as means with standard deviation in parentheses, and categorial variables are presented as n-numbers with percentage in parentheses. BMI (body mass index), D.m. II (diabetes mellitus type II), PAD (peripheral artery disease), ASA (American Society of Anesthesiologists physical status classification system), AEG (esophagogastric junctional adenocarcinoma), SCC (squamous cell carcinoma)

Parameter	Total, *n* = 138 (100)	Non-AL, *n* = 105 (76.1)	AL, *n* = 33 (23.9)	*p* value
Male	111 (80.4)	85 (76.6)	26 (23.4)	0.804
Age	62.8 (11.1)	61.9 (11.4)	65.7 (9.6)	0.064
BMI	25.4 (4.8)	25.7 (4.8)	24.6 (4.6)	0.258
Hypertension	77 (55.8)	54 (70.1)	23 (29.9)	0.073
D.m. II	18 (13.0)	15 (83,3)	3 (16.7)	0.562
pAVK	9 (6.5)	7 (77.8)	2 (22.2)	1.000
Coronary artery disease	26 (18.8)	16 (61.5)	10 (38.5)	0.073
Stroke	6 (4.3)	3 (50.0)	3 (50.0)	0.148
Heart insufficiency	26 (18.8)	17 (65.4)	9 (34.6)	0.201
ASA				0.123
* I*	29 (21.0)	26 (89.7)	3 (10.3)	
* II*	47 (34.1)	33 (70.2)	14 (29.8)	
* III*	62 (44.9)	46 (74.2)	16 (25.8)	
Diagnosis				0.053
* AEG I*	53 (38.4)	46 (86.8)	7 (13.2)	
* AEG II*	39 (28.3)	27 (69.2)	12 (30.8)	
* AEG III*	2 (1.4)	0	2 (100)	
* SCC*	35 (25.4)	25 (71.4)	10 (28.6)	
* Adenocarcinoma*	4 (2.9)	3 (75.0)	1 (25.0)	
* Neuroendocrine carcinoma*	2 (1.4)	1 (50.0)	1 (50.0)	
* Achalasia*	1 (0.7)	1 (100)	0	
* Leiomyoma*	1 (0.7)	1 (100)	0	
* Occlusion*	1 (0.7)	1 (100)	0	
Neoadjuvant therapy	120 (87.0)	94 (78.3)	26 (21.7)	0.110
Radiotherapy	31 (22.5)	22 (71.0)	9 (29.0)	0.448
T-stage				0.364
* 0*	12 (8.7)	8 (66.7)	4 (33.3)	
* 1*	29 (21.0)	23 (79.3)	6 (20.7)	
* 2*	30 (21.7)	19 (63.3)	11 (36.7)	
* 3*	60 (43.5)	49 (81.7)	11 (18.3)	
* 4*	4 (2.9)	3 (75.0)	1 (25.0)	
N-stage				0.962
* 0*	79 (57.2)	60 (75.9)	19 (24.1)	
* 1*	24 (17.4)	19 (79.2)	5 (20.8)	
* 2*	14 (10.1)	10 (71.4)	4 (28.6)	
* 3*	16 (11.6)	12 (75.0)	4 (25.0)	
M-stage				0.335
* 0*	126 (91.3)	94 (74.6)	32 (25.4)	
* 1*	6 (4.3)	6 (100)	0	

### Elevated serum lactate is associated with the incidence of AL after esophagectomy

To identify potential parameters associated with the incidence of AL, serum albumin (normal range: 3.5–5.0 g/dl), serum creatinine (normal range: 0.7–1.3 mg/dl), international normalized ratio (INR), and activated partial thromboplastin time (aPTT; normal range: 26–37 s) were assessed before surgery, at POD 0 after surgery and on POD 1. Moreover, levels of serum lactate (mmol/l) were extracted on POD 0 at the start (POD 0–1) and the end of surgery (POD 0–2) as well as on POD 1. Although level of serum lactate did not differ between AL and non-AL patients at start of operation [POD 0–1: AL vs. no AL: 0.9 (0.25) vs. 0.9 (0.31); *p* = 0.373], AL patients showed a higher serum lactate level at end of surgery [POD0-2: 1.4 (0.65) vs. 1.1 (0.53); *p* = 0.014] which was even present at POD 1 [1.2 (0.38) vs. 1.0 (0.37); *p* < 0.001] (Table 2). Interestingly, increase in serum lactate level between end (POD 0–2) and start of surgery (POD 0–1) (*p* < 0.001) as well as between POD 1 and start of surgery (POD 0–1) (*p* < 0.001) were significantly associated with the incidence of AL (Table 2). Furthermore, INR (*p* = 0.001) and aPTT (*p* < 0.001) on POD 1 were significantly associated with the occurrence of AL (Table 2). Patients with AL showed a significant prolongation of aPTT when compared to patients without AL on POD 1 [37.0 (5.69) vs. 32.0 (4.81); *p* < 0.001] (Table 2).

**Table 2:** Comparison between patients without and with AL. Data is presented as mean with standard deviation. AL (anastomotic leakage), POD (postoperative day), INR (international normalized ratio), aPTT (activated partial thromboplastin time). Serum albumin is depicted in g/dl, serum creatinine in mg/dl, aPTT in s, and serum lactate in mmol/l

	Al	No Al	*p* value
Preoperative albumin	4.5 (.58)	4.4 (.34)	.460
Albumin POD 0	3.0 (.74)	3.1 (.29)	.251
Albumin POD 1	3.1 (.61)	3.1 (.36)	.900
Preoperative creatinine	.09 (.26)	.09 (.20)	.442
Creatinine POD 0	.80 (.26)	.80 (.19)	.980
Creatinine POD 1	.80 (.31)	.80 (.22)	.780
Preoperative INR	1.0 (.08)	.9 (.07)	.087
INR POD 1	1.1 (.12)	1.1 (.97)	.001
Preoperative aPTT	30.0 (4.32)	29.0 (3.26)	.380
aPTT POD 1	37.0 (5.69)	32.0 (4.81)	< 0.001
Lactate POD 0–1	.9 (.25)	.9 (31)	.373
Lactate POD 0–2	1.4 (.65)	1.1 (.53)	.014
Lactate POD 1	1.2 (.38)	1.0 (.37)	< .001
Difference lactate POD 0–2 to POD 0–1	.50 (.61)	.10 (.49)	< .001
Difference lactate POD POD 1 to POD 0–1	.40 (.35)	.10 (.36)	< .001
Difference lactate POD 1to POD 0–2	.00 (.58)	-.10 (.48)	.927

### Serum lactate and aPTT independently predict the incidence of AL after esophagectomy

To further elucidate predictors of AL, parameters were subjected to logistic regression analysis. Here, serum lactate on POD 1 emerged as an independent predictor of AL with an HR of 4.37 (95% CI: 1.28–14.86) [*p* = 0.018] (Table 3). Furthermore, aPTT on POD 1 also predicted AL after esophagectomy (HR: 1.16 (95% CI: 1.06–1.26) [*p* = 0.001]) (Table 3). In contrast, INR on POD 1 and lactate POD 0–2 were no independent factors for AL with an HR of 0.83 (0.273–0.253) [*p* = 0.743] and 1.127 (0.512–2.49) [*p* = 0.766], respectively (Table 3).

**Table 3:** Independent predictors of anastomotic leakage. Data is presented as hazard ratio with a 95% confidence interval (CI). POD (postoperative day), INR (international normalized ratio), aPTT (activated partial thromboplastin time)

Logistic regression analysis	Hazard ratio	95%-CI	*p* value
INR POD 1	0.83	.273–2.53	.743
aPTT POD 1	1.16	1.06–1.26	.001
Lactate POD 0–2	1.127	.512–2.49	.766
Lactate POD 1	4.37	1.28–14.86	.018

### Low serum albumin is associated with in-hospital death after esophagectomy

In accordance to AL, serum level of albumin, lactate, and aPTT and INR were extracted to assess their impact on in-hospital death after esophagectomy.

Here, serum albumin on POD 0 was lower in non-survivors when compared with survivors [2.6 (0.53) vs. 3.1 (0.56); *p* = 0.001] (Table 4). Further, elevation of serum lactate at POD 0–1 was associated with in-hospital death [1.1 (0.29) vs. 0.9 (0.30); *p* = 0.043] (Table 4). Accordingly, preoperative INR [1.0 (0.06) vs. 0.9 (0.08); *p* = 0.014] as well as INR on POD 1 [1.2 (0.11) vs. 1.1 (0.90); *p* = 0.002] was elevated non-survivors when compared to the survivor group (Table 4). To this end, increased aPTT on POD 1 was significantly associated with in-hospital mortality [40.0 (5.52) vs. 33.0 (5.22); *p* = 0.027] (Table 4). In contrast, serum creatinine at no time point was associated with survival after esophagectomy (Table 4).

**Table 4:** Comparison between non-survivors and survivors. Data is presented as mean with standard deviation. POD (postoperative day), INR (international normalized ratio), aPTT (activated partial thromboplastin time)

	Non-survivor	Survivor	*p* value
Preoperative albumin	4.5 (.86)	4.4 (.35)	.842
Albumin POD 0	2.6 (.53)	3.1 (.56)	.001
Albumin POD 1	3.1 (.86)	3.1 (.38)	.910
Lactate POD 0–1	1.1 (.29)	.9 (.30)	.043
Lactate POD 0–2	1.4 (.67)	1.1 (.56(	.198
Lactate POD 1	1.4 (.50)	1.1 (.38)	.073
Difference lactate POD 0–2 to POD 0–1	.25 (.66)	.15 (.54)	.559
Difference lactate POD POD 1 to POD 0–1	.20 (.50)	.10 (.49)	.596
Difference lactate POD 1to POD 0–2	.05 (.47(	-.10 (.51)	.924
Preoperative INR	1.0 (.06)	0.9 (.08)	.014
INR POD 1	1.2 (.11)	1.1 (.90)	.002
Preoperative aPTT	28.0 (3.23)	29.0 (3.63)	.371
aPTT POD 1	40.0 (5.52)	33.0 (5.22)	.027
Preoperative creatinine	.90 (.27)	.90 (.21)	.478
Creatinine POD 0	.80 (.20)	.80 (.21)	.827
Creatinine POD 1	.80 (.26)	.80 (.24)	.483

### Serum albumin independently predicts survival after esophagectomy

**Fig. 2:**
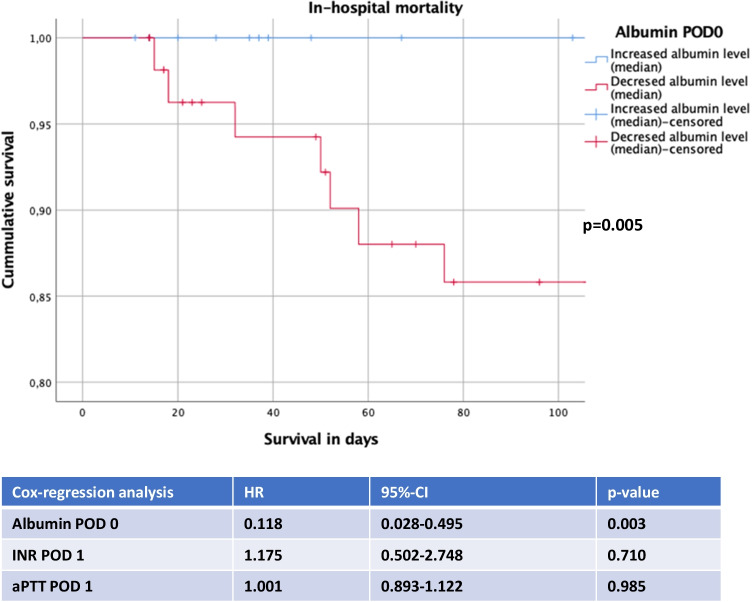
Cumulative survival. Data is presented as cumulative survival of patients after esophagectomy with the assessment of in-hospital death, depicted in a Kaplan–Meier analysis. POD (postoperative day), INR (international normalized ratio), aPTT (activated partial thromboplastin time)

To further elucidate predictors of in-hospital death, parameters were subjected to logistic regression analysis. The analysis revealed serum albumin POD 0 to be an independent predictor for survival after esophagectomy [HR: 0.111 (95% CI: 0.022–0.568); *p* = 0.008] (Table 5). On the other side, INR POD 1 [HR: 1.068 (0.457–2.498); *p* = 0.879] did not emerge as a predictor of survival after esophagectomy (Table 5). The impact of serum albumin on cumulative survival after esophagectomy was also reflected when comparing patients with increased and decreased median serum albumin level, the first showing a significantly improved in-hospital survival when compared to the latter group of patients (*p* = 0.005) (Fig. 2).

**Table 5:** Independent predictors of survival. Data is presented as hazard ratio with a 95% confidence interval (CI). POD (postoperative day), INR (international normalized ratio), aPTT (activated partial thromboplastin time)

Logistic regression analysis	Hazard ratio	95%-CI	*p* value
Albumin POD 0	.111	.022-.568	.008
INR POD 1	1.068	.457–2.498	.879
aPTT POD 1	1.008	.877–1.159	.909

## Discussion

Here, a single-center analysis to identify risk factors for the development of AL after esophagectomy and to elucidate predictors for in-hospital mortality was conducted. Serum lactate and aPTT on the first postoperative day were able to independently predict the incidence of AL after esophagectomy. Further, serum albumin at the time of resection emerged as an independent predictor of survival after resection.

AL is a severe complication after Ivor-Lewis esophagectomy, often leading to a prolonged hospital stay and increased mortality, as well as compromised long-term oncological outcomes [8, 21–23]. This is also reflected in a recent meta-analysis by Kamarajah et al. including 74,226 patients, where they found prolonged hospital stay (mean difference 15 days, *p* < 0.001) and increased in-hospital mortality (OR: 5.91, *p* = 0.015) in patients suffering from AL after esophagectomy [8]. Another study by Markar et al. investigated the impact of severe AL (postoperative complication grade III or IV according to Clavien-Dindo) on long-term survival and oncological outcome after esophagectomy [22]. In this multicenter study, of 2944 resected patients, 2439 patients were included in the final analysis. The rate of severe anastomotic leakage was 8.5%, and independent predictors were low hospital volume, cervical anastomosis, tumor stage III/IV, and cardiovascular as well as pulmonary complications [22]. They further demonstrated that severe anastomotic leakage was associated with a significant reduction in disease-free survival (34 months vs. 47.9 months; *p* = 0.005) as well as median overall survival (35.8 months vs. 54.8 months; *p* = 0.002) [22]. This was reflected by increased chance for locoregional (OR: 1.56; *p* = 0.030), mixed (OR: 1.81; *p* = 0.014), and overall (OR: 1.35; *p* = 0.011) cancer recurrence [22]. The likelihood of death was increased by 28% in patients with severe AL (OR: 1.28; *p* = 0.022) [22]. This negative impact of AL on oncological outcome has also been demonstrated for other GI cancers, like colorectal carcinoma [24]. Besides a hypothetical conduit for the spread of cancer cells at the site of AL, potentiating locoregional recurrence, also interleukins and cytokines within the septic condition of patients with AL might play a potential role and are discussed in the literature [25]. Together, these findings underline the pivotal importance of AL for the short-term as well as the long-term outcome of patients after esophagectomy. Further, it warrants adequate and timely treatment of AL, as the mechanism of poor outcome often involves the development of severe sepsis due to mediastinitis. Reports demonstrate a benefit from the early onset of treatment of AL using endoscopic vacuum therapy [15]. This underscores the importance of an independent predictor of AL within the postoperative course. This study identified both, serum lactate on postoperative day one and aPTT to predict the incidence of AL. Interestingly, also dynamics of serum lactate underscore its importance in the prediction of AL. Postoperative increase of serum lactate when comparing POD 1 with the start of operation as well as lactate dynamics during surgery (POD 0–2 vs. POD 0–1) reflect its pivotal relevance and the capability to monitor pathophysiological processes likely associated with ischemic events in the gastric tube within the perioperative phase of Ivor-Lewis-esophagectomy. These results are in line with findings of Ip et al., demonstrating serum lactate > 1.7 mmol/l on POD 2 to raise possibility for AL [26]. While the latter study found evidence for a possible association of serum lactate and AL, this study now was able to demonstrate by logistic-regression analysis, that serum lactate and aPTT were independent predictors of AL after Ivor-Lewis esophagectomy. Here, changes in aPTT might reflect compromised liver function or consumption of clotting factors within the perioperative setting. To this end, our study demonstrated serum albumin being an independent predictor of survival after esophagectomy. Low serum albumin has already been demonstrated to be associated with poor outcome in patients with squamous cell carcinoma, reflecting reduced nutritional and inflammatory status in those patients [27]. The present study underscored the importance of serum albumin not only in this group of patients, but also after resections due to other underlying diseases (e.g., AEG). Here, the recovery of albumin is considered a factor influencing inflammation and prognosis. Matsuda et al. showed that insufficient recovery after surgery confers a risk factor for systemic inflammatory response as well as poor prognosis [28]. In the present study, patients received a routine endoscopy on day 5 ± 3 after esophagectomy to evaluate anastomotic healing [19, 29]. If an AL was detected during endoscopy, patients received placement of an endoluminal vacuum sponge. Especially in asymptomatic patients, this held the potential of early and targeted therapeutic approach to AL. While being safe and having a high predictive value, this potential of intervention after early endoscopy was also demonstrated in a comparative study of Nishikawa et al. [30]. On the other hand, routine endoscopy is a field of discussion on the current literature and was not able to safely rule out the development of AL within the further course [31]. This underscores the importance to imply a multiple parameter in postoperative monitoring of AL.

The single-center nature and retrospective design, as well as the sample size and the mixed dignity (benign [*n* = 3] and malignant [*n* = 135]), confer a limitation to the study. On the other hand, the study provides a comprehensive analysis of perioperative blood results and profound statistical evaluation.

Taken together, this study demonstrated serum lactate and aPTT emerging as an independent predictor of AL after esophagectomy. This could facilitate early detection and treatment of AL, leading to improvement of short-term as well as long-term outcome of patients with Ivor-Lewis esophagectomy. Furthermore, serum albumin independently predicted in-hospital survival mandating for adequate reconstituting of serum albumin as well as nutritional status prior to resection.
